# Metabolic Dysfunction in the Regulation of the NLRP3 Inflammasome Activation: A Potential Target for Diabetic Nephropathy

**DOI:** 10.1155/2022/2193768

**Published:** 2022-06-09

**Authors:** Wenli Zhao, Le Zhou, Petr Novák, Xian Shi, Chuang Biao Lin, Xiao Zhu, Kai Yin

**Affiliations:** ^1^Department of Cardiology, The Second Affiliated Hospital of Guilin Medical University, Guilin, Guangxi, China; ^2^Guangxi Key Laboratory of Diabetic Systems Medicine, Guilin Medical University, Guilin, Guangxi, China; ^3^Guangxi Health Commission Key Laboratory of Glucose and Lipid Metabolism Disorders, The Second Affiliated Hospital of Guilin Medical University, Guilin, Guangxi 541199, China

## Abstract

Metabolic dysfunction plays a key role in the development of diabetic nephropathy (DN). However, the exact effects and mechanisms are still unclear. The pyrin domain-containing protein 3 (NLRP3) inflammasome, a member of the nod-like receptor family, is considered a crucial inflammatory regulator and plays important roles in the progress of DN. A growing body of evidence suggests that high glucose, high fat, or other metabolite disorders can abnormally activate the NLRP3 inflammasome. Thus, in this review, we discuss the potential function of abnormal metabolites such as saturated fatty acids (SFAs), cholesterol crystals, uric acid (UA), and homocysteine in the NLRP3 inflammasome activation and explain the potential function of metabolic dysfunction regulation of NLRP3 activation in the progress of DN via regulation of inflammatory response and renal interstitial fibrosis (RIF). In addition, the potential mechanisms of metabolism-related drugs, such as metformin and sodium glucose cotransporter (SGLT2) inhibitors, which have served as the suppressors of the NLRP3 inflammasomes, in DN, are also discussed. A better understanding of NLRP3 inflammasome activation in abnormal metabolic microenvironment may provide new insights for the prevention and treatment of DN.

## 1. Introduction

According to the statistics of the International Diabetes Federation (IDF) in 2019, the number of diabetes patients between the ages of 20 and 79 was expected to reach 578 million in 2030 [1]. One of the most serious consequences of diabetes is the development of diabetic vascular disease, which manifests clinically as microvascular and macrovascular complications [2]. Diabetic nephropathy (DN) is now one of the most serious microvascular complications of diabetes and is always accompanied by hyperglycemia, lipid metabolism disorder, oxidative stress, elevated advanced glycosylation end products (AGEs), etc. [3]. Although several available therapeutic interventions can delay the onset and progression of DN, the associated morbidity of this disease remains high due to its complex pathogenesis, suggesting that the novel therapeutic approaches are still needed to be explored.

Inflammasomes are a group of cytosolic protein complexes, which are formed to mediate host immune responses to cellular damage and microbial infection [4]. The pyrin domain-containing protein 3 (NLRP3) inflammasome is a classical inflammasome composed of NLRP3, adapter protein apoptosis-related speck-like protein (ASC), and the zymogen procaspase-1 [5]. Recent research has shown that the NLRP3 inflammasome plays an important role in various metabolic inflammatory diseases, such as atherosclerosis (As) and diabetes [6, 7]. NLRP3 monomers are assembled into cages and sense abnormal signals in the resting state [8]. The activation of the NLRP3 inflammasome, especially when stimulated by abnormal metabolites of glucose and lipids, can aggravate the maturation and secretion of proinflammatory cytokines (i.e., IL-1*β* and IL-18) and further trigger inflammatory cascades [9]. Furthermore, the activation of the NLRP3 inflammasome has been implicated in various pathological conditions, ranging from metabolic syndrome to kidney diseases [10]. Interestingly, preventing glomerular NLRP3 inflammasome activation by specific decrease in mitochondrial reactive oxygen species (ROS) by mitochondria-targeting antioxidant Mito-TEMPO can improve nephropathy in diabetic mice [11], suggesting that the NLRP3 inflammasome is a potential target in the treatment of metabolic inflammatory diseases, including renal injury in diabetes.

In this review, we discuss the exact roles of diabetic metabolic abnormalities in the activation of the NLRP3 inflammasome and summarize the underlying mechanism of NLRP3 inflammasome activation in the pathogenesis of DN. Moreover, a wide overview of the most promising metabolic drugs for the modulation of NLRP3 activation is also provided, which may offer new insights into the treatment of DN.

## 2. The Activation of the NLRP3 Inflammasome by Metabolite Abnormalities

**Figure 1:**
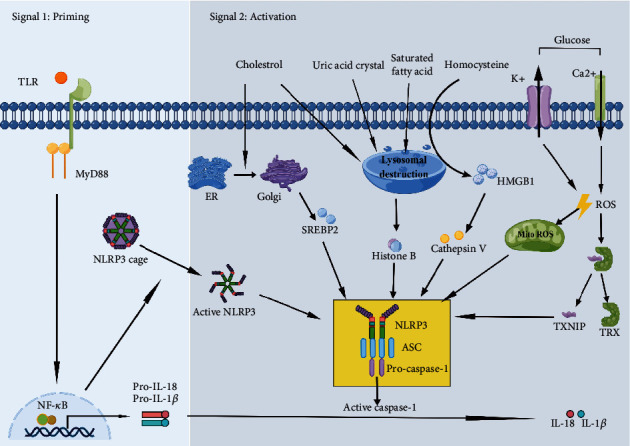
Specific mechanisms of NLRP3 inflammasome activation by abnormal accumulation of metabolites.

Diabetes is characterized by clustered metabolic abnormalities, such as hyperglycemia and elevated triglycerides [3]. In a diabetic kidney, specific metabolically induced glucose-dependent pathways are triggered, which induces oxidative stress, hexosamine flux, polyol pathway flux, and accumulation of AGEs [3]. Importantly, binding of AGEs to their receptor (RAGE) promotes the production of cytosolic ROS and stimulates intracellular signal molecules such as nuclear factor-*κ*B (NF-*κ*B) and protein kinase C (PKC), inducing the activation of transforming growth factor beta (TGF-*β*) and vascular endothelial growth factor (VEGF). Importantly, the metabolite abnormalities in DN can trigger the activation of the NLRP3 inflammasome. There is a dynamic mutual regulatory relationship between metabolism and inflammation, called the metabolic-inflammatory circuit [9]. Chronic inflammatory response increases the risk of insulin resistance in type 2 diabetes mellitus (T2DM). The association between the NLRP3 inflammasome and T2DM is increasingly accepted [12]. As such, we further explore how metabolite abnormalities regulate the NLRP3 inflammasome in kidney-related cells (Table 1 and Figure 1).

### 2.1. Glucose

The high blood sugar state caused by glucose metabolism disorder is the basic and necessary link of diabetes. In human cell models and in murine models of diabetes, hyperglycemia stimulated NLRP3 inflammasome activation, subsequently causing injury to pancreatic islet cells, glucose intolerance, and insulin resistance [13]. Therefore, we summarize these mechanisms. Most typically, high glucose can mediate external discharge of K^+^ and inward flow of Ca^2+^, which induces ROS overproduction and activation of the NLRP3 inflammasome in monocytes [14, 15]. When exposed to high glucose, ROS in mesangial cells increases expression of p38 and forkhead box protein O1 (FOXO1), and thioredoxin-interacting protein (TXNIP) separates from its conjugate with TRX and binds directly to NLRP3, inducing activation of the NLRP3 inflammasome assembly [16–19]. Also, NF-*κ*B is a nuclear transcription factor with enhanced transcriptional activity in high glucose states. It was recently found that the NLRP3 inflammasome is activated due to the direct interaction of p50 (a subunit of NF-*κ*B) with the NLRP3 promoter when exposed to high glucose [20]. In addition, hyperglycemia may contribute to the activation of the NLRP3 inflammasome mediated by pyruvate kinase M2 (PKM2) in macrophages [21]. In conclusion, NLRP3 is an important critical point between metabolism and inflammation. Currently, it has been demonstrated that epigenetic transcription (e.g., m6a) can trigger NLRP3 inflammasome activation [22] and high glucose can affect epigenetic transcription [23]. It remains to be further investigated whether high glucose affects NLRP3 by affecting epigenetic transcription.

### 2.2. Fatty Acids

Fatty acids (FAs) are one of the most abundant lipids in plasma, including saturated fatty acids (SFAs) (e.g., palmitic acid), monounsaturated fatty acids (MUFAs) (e.g., oleic acid), and polyunsaturated fatty acids (PUFAs) (e.g., omega-3FAs and omega-6FAs). SFA levels in plasma of patients with T2D on a high-fat diet were elevated [24]. SFAs, especially their crystals (e.g., palmitate), is known to directly influence inflammatory processes [25]. Specifically, palmitate can activate the NLRP3 inflammasome through lysosomal destabilization in macrophages [26]. Additionally, palmitate inhibits adenosine 5′-monophosphate-activated protein kinase (AMPK) phosphorylation and blocks autophagy, leading to increased levels of ROS in macrophages, which in turn activates the NLRP3 inflammasome and IL-1*β* secretion during T2D [27]. MUFAs can inhibit the NF-*κ*B and NLRP3 activation through direct binding to GPR120 (G protein-coupled receptor 120) or PPARs (peroxisome proliferator-activated receptors) and through AMPK phosphorylation [28]. SFA-induced NLRP3 activation can obviously be inhibited in the presence of MUFAs [29], indicating that the balance of SFAs and MUFAs is a critic point for NLRP3 inflammasome activation. Interestingly, it is controversial whether regular PUFA intake can be used as a pharmacological replacement therapy for diabetes. A double-blind randomized clinical trial showed that n-3 PUFAs improve glycemic control in Asians [30]. However, increasing PUFAs had little to no effect on the prevention and treatment of T2D, based on studies of randomized participants from around the world [31]. It is recommended that the protective effect of w-3 PUFAs on T2D may be influenced by ethnicity.

### 2.3. Cholesterol

Cholesterol is a multifunctional lipid that can be ingested from the diet or synthesized by the endoplasmic reticulum (ER). In patients with poorly controlled and/or insulin-resistant diabetes, both cholesterol production and cholesterol genesis are increased [32]. The accumulation of cholesterol can form crystals in lysosomes and further disrupt the lysosomal membrane and lysosomal stabilization after entering the cell [33]. Importantly, this destabilization can aggravate the release of histone B into the cytoplasm, which activates the NLRP3 inflammasome and causes the secretion of mature IL-1*β* [34]. Moreover, the redundant cholesterol in lysosomes can be transported to the ER and further stimulate the NLRP3 inflammasome [35]. In addition, Guo et al. showed that sterol regulatory element-binding protein 2 (SREBP2) cleavage-activating protein- (SCAP-) SREBP2 promotes NLRP3 inflammasome activation, which is largely dependent on cholesterol ER to Golgi translocation [36], indicating that interorganelle cholesterol mobility is essential for the activation of the NLRP3 inflammasome. However, it still needs to be explored why and how cholesterol, as an important regulator of the membrane integrity and fluidity, stimulates the NLRP3 inflammasome in different organelles.

### 2.4. Uric Acid

Uric acid (UA) is a purine metabolite that is produced in high quantities upon cellular injury [37]. Its level is affected by the amount of its production and reabsorption by the kidneys and intestines. Both clinical and epidemiological studies have confirmed that UA plays a vital role in the occurrence and development of insulin resistance, lipid metabolism disorders, and metabolic syndrome [38]. Cohort studies have shown that increased levels of UA are associated with the increased risk of diabetes and DN [39]. Mitochondrial ROS activated by high levels of UA mediates NLRP3 activation and IL-1*β* secretion and activates NF-*κ*B in cocultured macrophages and proximal renal tubular cells [40]. Notably, when UA exceeds the threshold, it precipitates out of different tissues and body fluids and forms crystals [41]. The elevated UA crystals can activate the NLRP3 inflammasome to trigger IL-1*β*-mediated inflammation by directly binding to the lipids on the surface of macrophages [42]. Interestingly, an earlier study also demonstrated that UA crystals induced the dissociation of TXNIP from thioredoxin (TRX) in the presence of ROS, allowing it to bind to NLRP3 and enhance caspase activation [43]. Furthermore, the synergistic effect between FFAs and urate crystals leads to activating the NLRP3 inflammasome [44], suggesting that different metabolites associated with diabetes interact with each other to promote the development of inflammation. With further development of metabolomic technologies, a deeper understanding of the currently known metabolite interaction pathways and possible mechanisms can be gained.

### 2.5. Homocysteine

Homocysteine (HCY), a sulfur-containing amino acid, is derived from protein catabolism. Elevated levels of plasma HCY (to more than 15 *μ*M, defined as hyperhomocysteinemia (HHCY)) are an independent risk factor in diabetes [45]. Recent findings demonstrated that the increased HCY in the blood can promote NLRP3 inflammasome formation by different mechanisms [46]. For example, HCY is involved in NLRP3 inflammasome and caspase-1 activation and increased vascular endothelial inflammation by raising high mobility group box-1 protein (HMGB1), lysosomal permeability, and lysosomal cysteine protease tissue proteinase V [47]. Additionally, in vascular smooth muscle cells (VSMCs), HCY stimulates NLRP3 inflammasomes through regulating extracellular regulated protein kinases1/2 (ERK1/2) and p38 MAPK pathways [48]. Furthermore, elevated levels of HCY have been found to activate the guanine nucleotide exchange factor Vav2 [49]. Other studies show that Vav2-mediated Rac1 GTPase activity can trigger NLRP3 inflammasome activation by leading to oxidative stress via increasing nicotinamide adenine dinucleotide (NADPH) oxidase activity [50, 51]. It is suggested that the role of HCY in NLRP3 activation partly relies on the Vav2-mediated pathway. Meanwhile, HHCY can increase oxidative stress and its downstream signaling pathway, so whether HHCY activates NLRP3 through oxidative stress activation pathway is worth exploring. HCY has also been shown to increase the hypoxia inducible factor-1*α* (HIF1*α*) protein levels [52]. Moreover, HIF-1*α* upregulates pla2g16 (a novel HIF-1*α* target gene) gene expression to activate the NLRP3 inflammasome pathway [53]. It is suggested that HCY may activate the NLRP3 inflammasome through hypoxia-related pathways. Besides, HCY induces inflammation in adipocytes in a manner that affects lipid status and causes NLRP3 activation [54], so it is worth exploring whether NLRP3 can be activated in other tissues and cells in a similar manner.

## 3. The Role of the Abnormal Metabolite-Induced NLRP3 Inflammasome Activation in DN

NLRP3 inflammasome-mediated inflammation is recently recognized in the development of kidney injury [56]. DN undergoes a transition from renal inflammation to fibrosis [57]. Renal NLRP3 overexpression is associated with macrophage infiltration and fibrosis [58]. When the microenvironment is altered, the kidney is in an acute kidney injury (AKI) state, and as the first defender, immune cells maintain cellular homeostasis. In a mild AKI, a renal tubular injury is fully recovered. Notably, a severe AKI becomes chronic with high levels of NLRP3 in serum or urine. This induces a glomerular injury affecting glomerular endothelial cells, thylakoid cells, and podocytes [20, 59, 60]. When the disease progresses further, dominant NLRP3 is predominantly distributed in abnormal renal tubules surrounded by inflammatory infiltration and fibrosis, and tubular epithelial cells are atrophied and dispersed, indicating maladaptive repair [58].

### 3.1. NLRP3-Mediated Inflammation in DN

#### 3.1.1. Immune Cells

Renal inflammation includes the release of cytokines and chemokines and infiltration of immune cells, and upregulation of inflammatory signaling pathways is involved in the development and progression of DN [61]. Evidence from clinical laboratory studies suggests that infiltration of immune cells (mainly macrophages) is commonly observed in the glomeruli and interstation of renal biopsy specimens at all stages of DN [62]. Overexpression of NLRP3 leading to elevated proinflammatory cytokines IL-1*β* and IL-18, followed by inflammatory cell infiltration in the glomerulus, was discovered in a study of diabetic nephropathy rats regarding hyperuricemia and dyslipidemia [56]. However, the results of a bone marrow transplantation study suggest that NLRP3 among renal nonhematopoietic cells plays a more important role than natural immune cells in mediating the inflammatory process of DN [63].

#### 3.1.2. Renal Resident Cells

The inflammasome activation is detected in podocytes and endothelial cells during the early stages of nephropathy in db/db mice [11]. In the kidneys of STZ-induced diabetic mice, hyperglycemia induces TXNIP expression, activates Nox to produce ROS, and subsequently triggers the inflammasome activation in podocytes leading to podocyte loss and albuminuria [64]. The inhibition of NLRP3 and ASC by shRNA inhibits the high glucose-induced activation of IL-1*β* expression and attenuates the podocyte injury [65]. As the disease progresses, the renal tubular injury becomes one of the key determinants of DN. The role of the NLRP3 inflammasome in the tubular injury has been confirmed in different studies. For example, in proximal renal tubular cells, the activation of the NLRP3 inflammasome by high glucose was also inhibited by the inhibition of the tyrosine protein kinase SYK, suggesting a role for SYK-JNK-NLRP3 signaling in the pathogenesis of DN [66]. Expression of optineurin (an autophagic receptor for damaged mitochondria during mitochondrial phagocytosis) during the process of mitophagy was reduced in tubular epithelial cells from patients with DN compared with those from nondiabetic healthy individuals and was negatively correlated with renal interstitial inflammation [67]. In mouse renal tubular epithelial cells, optineurin overexpression enhanced mitophagy and inhibited high glucose-induced NLRP3 expression, CASP1 cleavage, and IL-1*β* and IL-18 release [67]. Furthermore, ischemia, toxins, and albuminuria can cause tubulointerstitial inflammation, which can cause an extracellular matrix injury and further exacerbate tubulointerstitial inflammation [68]. A cycle between the extracellular matrix injury and inflammation can be formed, and regulating its balance may be essential for inhibiting the progression of renal fibrosis. Resident fibroblasts also display a more proinflammatory phenotype and actively drive the inflammatory response during renal injury [69].

### 3.2. NLRP3-Mediated Renal Fibrosis in DN

**Figure 2:**
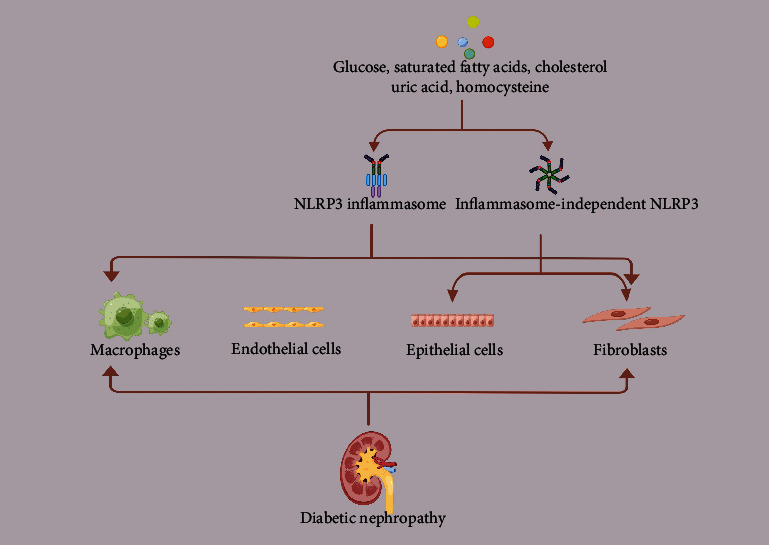
Role of inflammasome-independent NLRP3 and the NLRP3 inflammasome in the pathogenesis of diabetic nephropathy.

In essence, renal fibrosis is an integral pathological development in DN [70]. The main mechanisms involved in fibrosis are massive inflammatory cell infiltration [71], epithelial-mesenchymal transition (EMT) [72], endothelial-mesenchymal transition (EndoMT) [71], the activation of interstitial myofibroblasts [73], and the resulting accumulation of extracellular matrix components, which eventually replace the normal renal structure and form scarring, resulting in the loss of renal function (Figure 2).

### 3.3. Immune Cells

Immune cells have received much attention for their pathogenic role in renal fibrosis. The activation of IL-36 signaling in macrophages and dendritic cells positively regulates IL-1*β* secretion in a MyD88-dependent manner through NLRP3 inflammasome initiation signaling and promotes the development of kidney inflammation and fibrosis in mice [74]. Other evidence suggests that the inhibition of the NF-*κ*B-ROS-NLRP3 signaling pathway in the macrophage activation attenuates IgA progressive nephropathy and blocks glomerulosclerosis [75]. Moreover, the NLRP3 inflammasome activation in macrophages can promote chemokine signal transduction in the proximal tubule through intercellular crosstalk and eventually contribute to macrophage infiltration and tubulointerstitial fibrosis in the diabetic kidney [40].

### 3.4. Renal Resident Cells

A study showed the activation of the TLR4-NF-*κ*B-NLRP3 signaling pathway causing EMT and further transition to a fibrotic state [76]. Similarly, the role of the NLRP3 inflammasome in the tubular injury was demonstrated by the attenuation of high-glucose-induced EMT and inhibition of the phosphorylation of SMAD3, MAPK p38, ERK1, and ERK2 (key signaling molecules with roles in proinflammatory and profibrotic responses in tubular cells) in NLRP3-silenced HK-2 cells [77]. NLRP3 inflammasomes, an essential element of the innate immune response, are present in the progression of endothelial dysfunction associated with chronic kidney disease (CKD). Specifically, it was shown that the TLR4-Akt-NF-*κ*B-ROS-NLRP3 pathway contributes to inflammation-mediated endothelial dysfunction in CKD [78]. Since NLRP3 is involved in renal lesions, its involvement in the renal EndoMT process can be postulated. A study established the SIRT3-Foxo3a-Parkin pathway as a key factor in maintaining endothelial homeostasis and pointed to an important role of EndoMT in the vascular pathology of renal fibrosis [79]. Moreover, it was shown that the activation of the NLRP3 inflammasome in atherosclerosis via the SIRT3-SOD2-mtROS signaling pathway promotes inflammation in HUVECs [80]. Therefore, it deserves further investigation whether the NLRP3 activation induces renal EndoMT through SIRT3-related pathways. The aberrant activation and proliferation of fibroblasts are thought to be a key cause of renal fibrosis. Evidence exists that the inhibition of PERK-Akt-mTOR-NLRP3 signaling inhibits the renal fibroblast activation and fibroblast proliferation [81]. In addition to this, NF-*κ*B translocation and ROS production in renal thylakoid cells exposed to angiotensin II activate NLRP3 inflammasomes, which can lead to glomerular fibrosis [82]. Several studies have emphasized the importance of immunocyte activation, but it should be kept in mind that no single type of cells can initiate and sustain the overall renal fibrosis in isolation. Renal fibrogenesis explicitly necessitates the participation and interaction of many types of infiltrating cells, as well as resident kidney cells.

Moreover, NLRP3 has inflammasome-independent (noncanonical) effects leading to renal fibrosis in DN [83]. Inflammasome-independent NLRP3 in renal tubular cells plays an important role in the mitochondrial ROS injury by binding to mitochondrial antiviral signaling proteins after the hypoxic injury. In the absence of NLRP3, this mitochondrial regulation increases autophagy and attenuates renal tubular interstitial fibrosis [84]. Furthermore, NLRP3 promotes renal tubular EMT by enhancing TGF-*β*1 signaling and the R-Smad activation. The effect of NLRP3 on TGF-*β*1 signaling is independent of inflammasome components [83]. These data identify a novel inflammasome-independent and direct profibrotic role for NLRP3 in the renal tubular epithelium. Moreover, renal fibroblast inflammasome-independent NLRP3 also promotes fibrosis by enhancing TGF-*β* and Smad signaling without IL-1*β* production [74]. Thus, in the context of direct injury to renal tubular epithelial cells and fibroblasts, inflammasome-independent NLRP3 plays a key role in renal disease by regulating apoptosis, fibrosis, and the mitochondrial injury. This unique role of NLRP3 in the kidney can be clarified by conditional, cell type-specific regulation of the NLRP3 gene [85]. Activation of these signaling pathways leads to infiltration of circulating inflammatory cells, which amplifies and maintains the inflammatory process in the kidney and ultimately mediates or contributes to the diabetic renal fibrosis cascade response [86]. In addition to the proinflammatory cascade with NLRP3, the kinin-releasing enzyme-kinin system and protease-activated receptor signaling and the complement system (C5a and C3a) also play a role in fibrosis in diabetic kidney injury [61]. There are still a lot of underlying mechanisms waited to be further explored.

## 4. Metabolic Drugs Reverse the NLRP3 Inflammasome Activation in the Treatment of DN

Downregulation of inflammatory responses by therapeutic strategies can effectively prevent kidney disease development and improve renal function in patients with diabetes [87]. A growing body of evidence demonstrates that drugs reversing NLRP3 inflammasome activity have therapeutic potential for the treatment of DN, as discussed below (Table 2).

### 4.1. Metformin

Metformin is currently a first-line antidiabetic agent that reduces glucose through several different pathways: (i) inhibition of hepatic gluconeogenesis, (ii) improved insulin signaling through AMPK activation, (iii) enhanced peripheral insulin sensitivity due to increased glucose consumption, and (iv) induction of glucose transporter protein type 4 (GLUT-4) localization [88]. It has recently been suggested that some glucose lowering may be mediated through the enteroendocrine axis [89, 90]. Recently, there are some data showing that metformin exerts anti-inflammatory effects [91]. For example, upregulated NLRP3 inflammasome activation was found in macrophages collected from T2D patients and was downregulated after treatment with metformin [92]. A clinical randomized placebo-controlled study by Bhansali et al. underscores that in patients with T2D, metformin upregulated mitochondrial autophagy and subsequently improved alterations in mitochondrial morphology and function, independent of a hypoglycemic effect [93]. Then, further research is needed into whether metformin inhibits NLRP3 activation through mitochondrial autophagy. Furthermore, in APOE-/- male mice, metformin can reverse the decreased expression of thioredoxin-1, a stimulator of the NLRP3 inflammasome, which is induced by high glucose [94]. These studies hypothesized that metformin can partly treat diabetic kidney injury by combining with NLRP3 inflammasome-related multiple mechanisms, and whether new mechanism of this pathway exists deserves further investigation.

### 4.2. SGLT 2 Inhibitors

Sodium glucose cotransporter 2 inhibitors (SGLT-2is) reduce plasma glucose and hemoglobin A1c (HbA1c) levels in patients with T2D by increasing glucose excretion through inhibition of the proximal renal tubular reabsorption segment [95]. SGLT-2is, including dapagliflozin, ertugliflozin, and empagliflozin, are commonly used as clinical drugs. Kawanami et al. have demonstrated that SGLT-2is attenuate DN in diabetes animal models, suggesting a potential renal protective effect in addition to glucose reduction [96]. Recently, in a systematic evaluation and meta-analysis of clinical cardiovascular trials, exploratory results have shown that drugs such as SGLT-2is improve renal regression in patients with T2D [95, 97]. With in-depth studies, the inhibition of the NLRP3 inflammasome comes to the fore role in the process of SGLT 2 treatment [98]. For example, T2D patients treated with dapagliflozin showed reduced IL-1*β* secretion with increased serum *β*-hydroxybutyrate (BHB) and reduced serum insulin, and the inhibitory effect of both on NLRP3 inflammasome activation was verified in vitro [99]. Similarly, Benetti et al. demonstrated that empagliflozin significantly reduces diabetic renal NLRP3 inflammasome activity and attenuates downstream inflammatory responses [100]. In conclusion, although more research is needed, SGLT-2is appear to exert anti-inflammatory effects by inhibiting NLRP3 inflammasome activity, thereby benefiting the diabetic kidney.

### 4.3. DPP4 Inhibitors

Dipeptidyl peptidase-4 (DPP4) is a family member of serine proteases. DPP4 inhibitors (DPP-4is) exert hypoglycemic effects by inhibiting the release of DPP4 and glucagon, which in turn leads to increased release of insulin secretion and elevated circulating insulin levels [101]. Sitagliptin, linagliptin, saxagliptin, alogliptin, and vildagliptin are DDP-4is that can be used alone or in combination with other types of antidiabetic drugs. For example, many meta-analyses have found that in patients with T2D without adequate insulin control, DPP-4is show better glycemic control compared to placebo [102, 103]. Meanwhile, in another meta-analysis, the addition of DPP-4is in patients with T2D with inadequate alpha-glycosidase inhibitor (AGI) control resulted in better glycemic control [104]. More recently, studies have shown that DPP-4is can be used to fight inflammatory kidney damage caused by diabetes [105]. Birnbaum et al. found that saxagliptin reduces kidney injury and prevents DN progression by inhibiting NLRP3 in diabetic mice [106]. Similarly, combination of DPP-4i and SGLT-2i reduces NLRP3/ASC inflammasome activation and attenuates the development of diabetic nephropathy in type 2 diabetic mice [105]. More clinical research is needed to determine the role of DPP-4i in diabetic kidney injury. Currently oral DPP-4is do not reduce adipose inflammation or improve insulin resistance. Meanwhile, an article reported that intrahepatocellular but not intestinal DPP4 reduces adipose inflammation and improves insulin resistance while lowering blood glucose [107, 108]. Therefore, it could be considered that DPP-4i drugs could be redirected by packaging them into nanoparticles delivered to the liver, or attaching siRNAs to certain sugar molecules with specific affinity for hepatocytes could be a potential new target for the treatment of T2D and metabolic diseases.

### 4.4. Resveratrol

**Figure 3:**
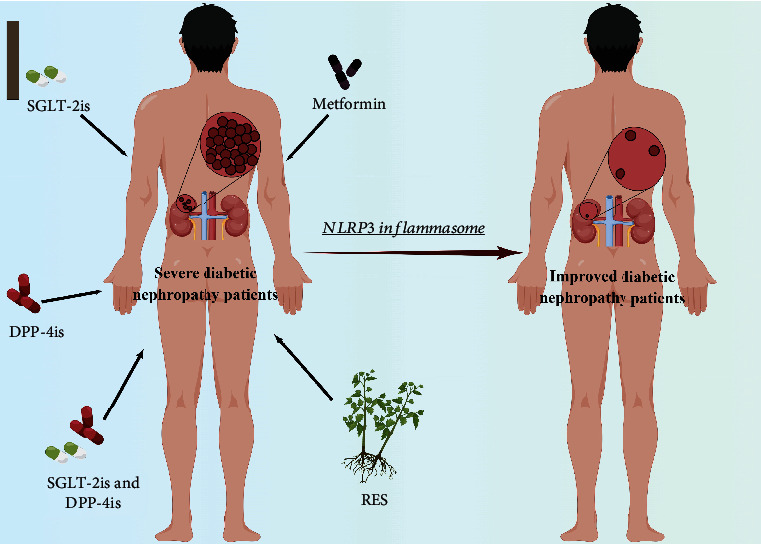
Clinical agents targeting NLRP3 inflammasomes for the treatment of diabetic nephropathy.

Resveratrol (3,5,4′-trihydroxy-trans-stilbene; RES) is a highly concentrated natural plant polyphenol found in red grapes and is also abundant in knotweed, soybeans, peanuts, and mulberries [109]. RES is known to have antioxidant, anticancer, antiobesity, anti-inflammatory, and antiaging effects [110]. Furthermore, current clinical trials have shown that RES also has antihyperglycemic effects [111, 112]. For example, resveratrol has been shown not only to lower blood glucose levels and protect *β*-cells in patients with type 1 diabetes [113] but also to improve insulin sensitivity in patients with T2D [114]. Notably, a study has shown that anti-inflammatory effects of RES may play a kidney-protective role in different diseases, including in diabetes [115]. Saldanha et al. show that RES inhibits or counteracts NF-*κ*B activity and coordinates the inflammatory response, thereby improving CKD [116]. More importantly, RES administration attenuated glomerulosclerosis and inflammation, and these were associated with reduced renal mononuclear leukocyte infiltration and inhibition of renal NLRP3 inflammasome activation in progressive IgA nephropathy in mice [117]. Similarly, RES treatment significantly inhibited oxidative stress in diabetic rats with renal I/R injury undergoing TXNIP-mediated NLRP3 activation [118]. However, the biological effects of RES are greatly limited by its low water solubility, poor stability, and rapid metabolism in vivo. Therefore, it is important to consider whether advanced technologies such as nanoparticles can be used to improve pharmacokinetics, achieve targeted drug delivery, improve drug utilization, achieve sustained inhibitory effects on the NLRP3 inflammasome, and ameliorate diabetic kidney injury. It also remains to be confirmed whether the effect of different RES doses on inflammation affects these results in terms of efficacy and safety (Figure 3).

### 4.5. IL-22

IL-22, an important member of the IL-10 family, is a key cytokine that regulates tissue responses during inflammation [119]. The downregulation of IL-22 in vivo has recently been recognized as a risk factor for diabetes [120]. Clinical research shows that plasma IL-22 levels were negatively and dose-dependently associated with the prevalence of T2D in Chinese urban adults [121]. IL-22 gene therapy significantly reduced hyperglycemia and metabolic disorders in diabetic rats. In this context, it has also been investigated whether IL-22 has a therapeutic effect on diabetic kidney injury [122]. Notably, Wang et al. show that IL-22 gene therapy significantly reversed the renal activation of NLRP3 to exert anti-inflammatory functions in DN rats [122]. Clinically, IL-22 gene therapy significantly reduced renal fibrosis and proteinuria excretion in DN 138. Furthermore, Shen et al. developed a novel antivascular endothelial growth factor B (VEGFB)/IL22 fusion protein that was found to improve the inflammatory response associated with NLRP3 and reduce renal lipid accumulation in diabetic patients [123]. Although more clinical studies are needed, IL-22 can be predicted to have great potential in DN therapy in targeting NLRP3 inflammasome activation. Moreover, investigators can focus on the therapeutic opportunities of IL-22 and its involved metabolic regulation in various diabetic kidney diseases.

### 4.6. Other Drugs

In addition to the above-mentioned drugs, other drugs with potential effects on the upstream activation and downstream transduction mechanisms of the NLRP3 inflammasome are currently being explored. TXNIP is an upstream partner to NLRP3, and the association between them is necessary for downstream inflammasome activation [43]. Tan et al. used TXNIP deoxyribozyme (DNAzyme) to restrain the expression of TXNIP, subsequently downregulating the level of NLRP3 in the renal tubule interstitium of diabetic rats [124]. Two other studies show that sarsasapogenin (Sar), a steroidal sapogenin, markedly constrains the activation of NLRP3 in the renal cortex to play a protective role in diabetic rats [125, 126]. Similarly, Shi et al. used dabrafenib to inhibit receptor-interacting protein kinase-3 (RIPK3), which has been implicated as a regulator of NLRP3 inflammasome signaling. The dabrafenib-induced RIPK3 deficiency alleviates diabetes-induced renal fibrosis, in association with reduced activation of the NLRP3 inflammasome [127]. Additionally, since there is crosstalk between metabolism and inflammation, researchers have attempted to inhibit NLRP3 inflammasome activation by improving metabolic pathways. Quercetin and allopurinol also repress renal NLRP3 inflammasome activation, at least partly, via their antihyperuricemia and antidyslipidemia effects, leading to the amelioration of STZ-induced superimposed nephrotoxicity in rats [56]. In addition, dihydroquercetin (DHQ) [128], catalpol [129], pioglitazone (PIO) [130], and curcumin [131] were also found to possess kidney protection effects associated with inhibiting NLRP3 activation in diabetic mice. These evidences suggest that targeting the NLRP3 inflammasome in DN may serve as a beneficial strategy for treatment.

## 5. Conclusions and Future Perspectives

It has become apparent that inflammation is an important element to initiate diabetic microvascular complications, including DN. Activation of the NLRP3 inflammasome is critical for the development of many kidney disorders including CKD, IgA nephropathy, lupus nephritis, and more. However, the onset and offset of these diseases are different due to their etiology and pathological features, but all have sustained NLRP3 inflammasome activation during the disease process, and inhibition of NLRP3 inflammasomes and related pathways may be a convergent strategy for the treatment of many renal diseases. Future research should focus on whether NLRP3 regulates the release of other mediators and thus exacerbates inflammation.

With the advent of epigenetics, it was found that not only can NLRP3 inflammasomes trigger epigenetic alterations [132] but also ShMETTL3 as used by Chien et al. blocks NLRP3 upregulation [133]. These data suggest a possible bidirectional feedback regulatory mechanism between epigenetic modifications and NLRP3-related inflammation. Additionally, red raspberry polyphenols were found to attenuate high-fat diet-induced NLRP3 inflammasome activation and inhibit adipogenic paracrine secretion through histone modifications [134], suggesting that NLRP3 may be a key hub bridging genetics and epigenetics. Now it needs to be further addressed which cell types of NLRP3 inflammasome activation can induce epigenetic reprogramming and further affect the physiological and pathological processes of the organism. The rise of single-cell sequencing able to reveal cellular heterogeneity in cell populations provides a desirable solution for this purpose.

Meanwhile, almost all epigenetic modification processes require the participation of metabolites, and the spatial regionalization of metabolites reveals the importance of metabolic enzyme translocation in epigenetic regulation. Therefore, discovering the role of metabolites in other organelles, such as lysosomes, the endoplasmic reticulum, and the Golgi apparatus, will be the key to understanding how metabolism and epigenetics interact and how organelles interoperate. Research has shown that the small molecule natural product xanthone can inhibit NLRP3 inflammasomes by configuring the cellular metabolic profile, leading to changes in glucose metabolism [135]. Therefore, systematic screening can identify novel NLRP3 inflammasome inhibitors to obtain new bioactive substances as potential drugs.

## Figures and Tables

**Table 1:** Aberrant metabolites activate the NLRP3 inflammasome in kidney-associated cells.

Stimulus	Kidney-related cells	Mechanism	Ref.
Glucose ↑	Monocytes	K^+^ outflow, Ca^2+^ inward flow/ROS/NLRP3 inflammasome	[14, 15]
	Glomerular mesangial cells	ROS/p38/FOXO1/TXNIP/NLRP3	[17–19]
		P50(NF-*κ*B)/NLRP3 inflammasome	[20]
	Macrophages	PKM2/NLRP3 inflammasome	[21]
Saturated fatty acids ↑	Macrophages	Lysosomal destabilization/NLRP3 inflammasome	[26]
		AMPK/ROS/NLRP3 inflammasome	[27, 55]
Cholesterol ↑	Macrophages	Lysosomal destabilization/histone B/NLRP3 inflammasome/IL-1*β*	[34]
		ER to Golgi translocation/SREBP2/NLRP3 inflammasome	[35, 36]
Uric acid ↑	Macrophages	ROS/NLRP3/IL-1*β*/NF-*κ*B	[40]
	Macrophages	ROS/TXNIP/NLRP3/caspase	[43]
Homocysteine ↑	Vascular endothelial cells	HMGB1/cathepsin V/NLRP3/caspase-1	[47]

ROS: reactive oxygen species; TXNIP: thioredoxin-interacting protein; FOXO1: forkhead box protein O1; NF-*κ*B: nuclear factor kappa B; PKM2: pyruvate kinase M2; AMPK: adenosine 5′-monophosphate-activated protein kinase; ER: endoplasmic reticulum; SREBP2: sterol regulatory element-binding protein 2; HMGB1: high mobility group box-1 protein; HIF1*α*: hypoxia inducible factor-1*α*; PDK1: 3-phosphoinositide-dependent kinase-1.

**Table 2:** The partly regulation role of drugs that target the NLRP3 inflammasome in DN treatment.

Medication	Related mechanisms	Experimental subjects	Ref.
Metformin	Inhibiting the NLRP3 inflammasome Improving phagocytosis, morphology, and function	Macrophages in T2D patients, APOE-/- male mice	[92–94]
SGLT-2is	Inhibiting the NLRP3 inflammasome/IL-1*β* axis	T2D patients	[99, 100]
DPP-4is DPP-4is and SGLT-2is	Inhibiting the NLRP3 inflammasome Inhibiting the NLRP3/ASC inflammasome	T2D patients, diabetic mice T2D mice	[106] [105]
RES	Inhibiting the TXNIP/NLRP3 axis	Mice with IgA nephropathy, diabetic rats with renal I/R injury	[116–118]
IL-22	Inhibiting the NLRP3/caspase-1/IL-1*β* axis	T2D in Chinese urban adults, diabetic patients, DN mice	[122, 123]
TXNIP DNAzyme	Inhibiting the TXNIP/NLRP3 axis	DN rats	[124]
Sar	Inhibiting NLRP3	DN rats	[125, 126]
Dabrafenib	Inhibiting the RIPK3/NLRP3 inflammasome axis	DN rat model	[127]
Quercetin and allopurinol	Inhibiting the caspase-1/IL-1*β*/IL-18 axis	STZ-induced DN	[56]
DHQ	Inhibiting the ROS/NLRP3 inflammasome	DN rats	[128]
Catalpol	Inhibiting ASC/NLRP3/caspase-1/IL-1*β*	DN mice	[129]
PIO	Inhibiting NLRP3/caspase-1/IL-18/IL-1*β*	DN mice	[130]
Curcumin	Inhibiting caspase-1/NLRP3	DN mice	[131]

SGLT2: sodium glucose cotransporter 2; DPP4: dipeptidyl peptidase-4; RES: resveratrol; Sar: sarsasapogenin; DHQ: dihydroquercetin; PIO: pioglitazone; TXNIP: thioredoxin-interacting protein.

## Abbreviations

DN: Diabetic nephropathy
NLRP3: Nucleotide-binding oligomerization domain leucine-rich repeat and pyrin domain-containing protein 3
SFAs: Saturated fatty acids
UA: Uric acid
RIF: Renal interstitial fibrosis
SGLT 2: Sodium glucose cotransporter 2
IDF: International Diabetes Federation
AGEs: Advanced glycosylation end products
ASC: Apoptosis-associated speck-like protein
As: Atherosclerosis
ROS: Reactive oxygen species
NF-*κ*B: Nuclear factor *κ*B
T2D: Type 2 diabetes
PKC: Protein kinase C
TGF-*β*: Transforming growth factor beta
VEGF: Vascular endothelial growth factor
TXNIP: Thioredoxin-interacting protein
FOXO1: Forkhead box protein O1
PKM2: Pyruvate kinase M2
MUFAs: Monounsaturated fatty acids
PUFAs: Polyunsaturated fatty acids
AMPK: Adenosine 5′-monophosphate-activated protein kinase
GPR120: G protein-coupled receptor 120
PPARs: Peroxisome proliferator-activated receptors
ER: Endoplasmic reticulum
SREBP2: Sterol regulatory element-binding protein 2
SCAP: Cleavage-activating protein
TRX: Thioredoxin
HCY: Homocysteine
HMGB1: High mobility group box-1 protein 1
VSMCs: Vascular smooth muscle cells
ERK1/2: Extracellular regulated protein kinase 1/2
NADPH: Nicotinamide adenine dinucleotide
HIF1*α*: Hypoxia inducible factor-1*α*
PDK1: 3-Phosphoinositide-dependent kinase-1
GFB: Glomerular filtration barrier
AKI: Acute kidney injury
GEnCs: Glomerular endothelial cells
EMT: Epithelial-mesenchymal transition
EndoMT: Endothelial-mesenchymal transition
CKD: Chronic kidney disease
DAMPs: Damage-associated molecular patterns
PAMPs: Pathogen-associated molecular patterns
GLUT-4: Glucose transporter protein type 4
HbA1c: Hemoglobin A1c
BHB: Serum *β*-hydroxybutyrate
DPP4: Dipeptidyl peptidase 4
DPP-4i: Dipeptidyl peptidase-4 inhibitors
AGI: Alpha-glycosidase inhibitor
RES: Resveratrol
VEGFB: Vascular endothelial growth factor B
DNAzyme: Deoxyribozyme
Sar: Sarsasapogenin
RIPK3: Receptor-interacting protein kinase-3
DHQ: Dihydroquercetin
PIO: Pioglitazone.
